# Synthesis, structure and luminescence studies of Eu(III), Tb(III), Sm(III), Dy(III) cationic complexes with acetylacetone and bis(5-(pyridine-2-yl)-1,2,4-triazol-3-yl)propane^☆^

**DOI:** 10.1016/j.ica.2013.04.006

**Published:** 2013-09-01

**Authors:** Alexey N. Gusev, Miki Hasegawa, Tomohito Shimizu, Tomonori Fukawa, Shoya Sakurai, Galyna A. Nishchymenko, Victor F. Shul’gin, Svetlana B. Meshkova, Wolfgang Linert

**Affiliations:** a Taurida National V.I. Vernadsky University, Simferopol 95007, Ukraine; b Department of Chemistry and Biological Science, College of Science and Technology, Aoyama Gakuin University, Kanagawa 252-5258, Japan; c A.V. Bogatsky Physico-Chemical Institute of the National Academy of Sciences of Ukraine, Odessa 65080, Ukraine; d Institute of Applied Synthetic Chemistry, Vienna University of Technology, Getreidemarkt 9/163, A-1060 Vienna, Austria

**Keywords:** Ln complex, 1,2,4-Triazole derivatives, X-ray study, Luminescence

## Abstract

Studies concerning synthesis, structure and luminescence of eight-coordinate Eu, Tb, Sm and Dy complexes of the type [Ln(acac)_2_(L)]Cl (Hacac = pentanedione-2,4 and L = bis(5-(pyridine-2-yl)-1,2,4-triazol-3-yl)propane) are reported in detail. The obtained complexes were investigated by various means including elemental- and thermogravimetric analysis, IR- and electron transition spectroscopy. The structure of the Tb complex was determined by single-crystal X-ray crystallography: Tb is eight-coordinate, and L acting only as a tetradentate chelate together with two bidentate acac ligands. Photophysical studies of the complexes were carried out. The Tb(III) and Eu(III) complexes show strong emissions both in solid state and solution. The intensity of the luminescence of Dy(III) and Sm(III) are relatively weak. The factors determining the intensity of the photoluminescence are discussed.

## Highlights

• Synthesis and structure of Ln(III) heteroleptic complexes using bistriazole ligands.
• Lifetime and quantum yield of luminescence are measured and discussed.
• Various factors determining the efficiency of luminescence were examined.

## Graphical abstract

New cationic heteroleptic Ln(III) complexes with acetylacetonate and bis(5-(pyridine-2-yl)-1,2,4-triazol-3-yl)propane basis are synthesized. The complexes are characterized by various means, including single crystal X-ray diffraction analysis. Electronic absorption and emission spectra of the compounds both in solid and solutions are studied.

**Figure:**
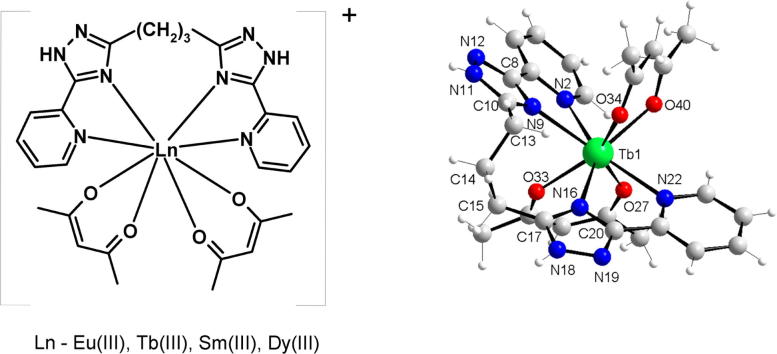

## 1 Introduction

In recent years the luminescence properties of rare earth metal complexes with various organic ligands have been widely studied. Especially Ln(III) complexes attract increasing attention due to numerous applications ranging from bio-medical diagnostics to photonic devices and solar energy conversion [1], [2], [3], [4], [5]. To obtain luminescent lanthanide complexes, the lanthanide ions require a suitable ligand acting as “antenna” and therefore as sensitizer for energy transfer in order to achieve efficient excitation of the metal ion. Additionally it should shield the central ion against the solvent in order to avoid non-radiative deactivation processes. The design of the suitable organic ligands plays a crucial role in the optimization of the photophysical properties of the lanthanide complexes for specific applications [6], [7], [8], [9]. Chemical modifications of ligands are obviously based on the problems to be solved: for example the creation of electroluminescent devices, which require predominantly molecular complexes soluble in nonpolar solvents. For bio-medical diagnostics one of the crucial requirements is solubility in aqueous media, which is provided by the introduction of polar substituents in the ligand or the synthesis of ionic complexes.

In previous articles a series of new luminescent Ln(III) complexes with pyridyltriazole derivatives as ancillary ligands [10], [11] have been described. These complexes exhibit significant luminescence intensity in solid state and in non-polar solvents. However, low solubility in water did not allow for a study of their luminescence in aqueous media. This encouraged us to study the synthesis, structure and luminescent properties of new cationic complexes [Ln(acac)_2_L]Cl where Ln(III) – Tb(III) (**1**), Eu(III) (**2**), Dy(III) (**3**) and Sm(III) (**4**); L – 1,3-bis(5-(pyridine-2-yl)-1,2,4-triazol-3-yl)propane which are reported in the present paper.

## 2 Experimental

### 2.1 Materials and methods

All solvents employed in this study were either AR or spectroscopic grade. Water was purified by the Milli Q system. The commercially available Ln_2_O_3_ (Ln = Sm, Eu, Dy; 99.9%) and Tb_4_O_7_ from Aldrich were converted to the corresponding chlorides, by the standard procedure. 1,3-Bis(5-(pyridine-2-yl)-1,2,4-triazol-3-yl)propane was prepared according to literature [10].

Elemental analyses of C, H, and N were performed with a Perkin–Elmer 240 C analyzer. IR spectra were measured with a Nicolet Nexus 470 FT-IR spectrometer with KBr pellets in the range 4000–400 cm^−1^. Thermal stability (TG-DTA) studies were carried out with a Paulik–Paulik–Erdey Q-derivatograph. Electronic spectra were recorded on a Perkin–Elmer Lambda-9 UV–Vis/NIR spectrophotometer in MeOH solution (10^−4^ M). Solid-state excitation and fluorescence spectra were recorded on a Horiba Jobin–Yvon Fluorolog-FL 3-22 spectrophotometer equipped with a 450 W Xe lamp. The luminescence quantum yield of solutions of the Ln complexes were determined by a comparison of the integrated corrected emission spectrum of standard quinine, which was excited at 366 nm in 0.1 M H_2_SO_4_ (*Q* = 0.55). For solid samples the quantum yields were determined under ligand excitation using an absolute method employing a home-modified integrating sphere. Time-resolved luminescence decay measurements were carried out on a Horiba Fluorocube lifetime instrument by a time-correlated single-photon counting method using a 365 nm LED excitation source.

### 2.2 Synthesis of complexes

A mixture of 400 mg (4 mmol) acetylacetone and 664 mg (2 mmol) L was dissolved in 10 ml of anhydrous MeOH. A solution of LnCl_3_ (2 mmol) in water was added drop wise to the reaction mixture. After 30 min 5 mmol of Et_3_N in 5 ml MeOH were added. The resulting mixture was stirred at room temperature for 3 h. Slow evaporation yields a polycrystalline solid, which was recrystallized, from MeOH. The pure complexes were collected by vacuum filtration and washed with methanol to provide colorless solids.

Single crystals of **1** fit for X-ray diffraction analysis were grown by recrystallization from a cyclohexane–MeOH mixture.

For 1: Yield: 438 mg, (60%). *Anal*. Calc. for C_27_H_30_ClO_4_N_8_Tb: C, 44.73; H, 4.17; N, 15.46. Found: C, 44.55; H, 4.83; N, 15.82%. IR (KBr, сm^−1^): *ν* = 3248 (m); 1596 (s), (C=O); 1518 (s), (C=N); 1454 (s); 1398 (s);1260 (m); 1016 (m); 921(m); 754 (m).

For 2: Yield: 523 mg, (73%). *Anal*. Calc. for C_27_H_30_ClEuO_4_N_8_: C, 45.16; H, 4.21; N, 15.61. Found: C, 44.98; H, 4.29; N, 15.34%. IR (KBr, сm^−1^): *ν* = 3246 (m); 1596 (s), (C=O); 1516 (s), (C=N); 1456 (s); 1397 (s); 1260 (m); 1016 (m); 924 (m); 752 (m).

For 3: Yield: 373 mg, (51%). *Anal*. Calc. for C_27_H_30_ClDyO_4_N_8_: C, 44.51; H, 4.15; N, 15.38. Found: C, 44.85; H, 4.33; N, 15.17%. IR (KBr, сm^−1^): *ν* = 3240 (m); 1597 (s), (C=O); 1519 (s) (C=N); 1456 (s); 1401 (s); 1262 (m); 1018 (m); 922 (m); 754 (m).

For 4: Yield: 582 mg, (81%). *Anal*. Calc. for C_27_H_30_ClO_4_N_8_Sm: C, 45.26; H, 4.22; N, 15.64. Found: C, 44.91; H, 4.18; N, 15.55%. IR (KBr, сm^−1^): *ν* = 3248 (m); 1596 (s), (C=O); 1518 (s), (C=N); 1456 (s); 1397 (s); 1260 (m); 1018 (m); 924 (m); 752 (m).

### 2.3 X-ray crystallography

An experimental array of reflection was obtained by the standard method. X-ray structural data were collected on a Rigaku VariMax with Saturn CCD diffractometer equipped with a monochromatic radiation source (Mo Kα radiation, *λ* = 0.71075 Å). The structure of 1 was solved by a direct method and refined via full-matrix anisotropic approximation for all non-hydrogen atoms. Hydrogen atoms of the carbon-containing ligands were geometrically generated and refined in the riding model. All calculations were performed using the Crystal Structure software package (rigaku). The crystallographic parameters and X-ray diffraction experimental-parameters are given in Table 1.

**Table 1:** Crystal data and experimental details for complex **1**.

Parameter	**1**
Formula	C_39_H_44_TbO_4_N_8_Cl
*T* (K)	296
Crystal system	triclinic
Space group	$P\overset{¯}{1}$
Unit cell parameters	
*а* (Å)	9.229(5)
*b* (Å)	13.790(11)
*c* (Å)	14.984(9)
*V* (Å)	86.64(5)
*α* (°)	72.50(4)
*β* (°)	88.62(5)
*γ* (°)	1816(2)
*Z*	2
*D*_calc_ (g сm^−3^)	1.518
*_μ_*_Mo_ (mm^−1^)	2.066
*F*(0 0 0)	844.00
*θ*_max._ (°)	2.74–25.99
Index ranges	−11 ⩽ *h* ⩽ 11, −17 ⩽ *k* ⩽ 17, −18 ⩽ *l* ⩽ 18
Reflections measured/reflections independent	41 519/7118 (*R*_int_ = 0.0378)
Parameters	440
Goodness-of-fit (GOF)	1.119
*R*(*I* > 2*σ* (*I*))	*R*_1_ = 0.0433, *wR*_2_ = 0.1074
Residual electron density (maximum/minimum) (е A^3^)	−1.21 and 2.59

## 3 Results and discussion

### 3.1 Synthesis of the complexes and general characterization

The ditopic ligand bis(5-(pyridine-2-yl)-1,2,4-triazol-3-yl)propane was obtained by the two-step reaction between pyridine-2-carbonitrile and the glutaric acids hydrazide. Presence of the mobile acidic hydrogen atom in triazole rings allows obtaining complexes both in their cationic and molecular forms. Last coordination mode was described for Eu(III) complexes [10]. Reaction of two equivalents acetylacetone and one equivalent of L with one equivalent of the respective Ln(III) salt in methanol–water solution and in the presence of 2.5 equivalents of triethylamine leads to coordination (5-(pyridine-2-yl)-1,2,4-triazol-3-yl)propane in cationic form. Slow evaporation of the reaction mixture yields colorless polycrystalline solids. The complexes have been characterized by IR- spectroscopy, elemental analysis and X-ray diffraction (for Tb complex). They are soluble in organic solvents and in water. TGA-DTA analyses of all the Ln(III) complexes were carried out and no solvent molecules were found. Considering photophysical properties, elemental and thermogravimetric analysis, IR spectroscopy of complexes **1**–**4** yield analog molecular structures.

### 3.2 Crystal structures of complex **1**

**Fig. 1:**
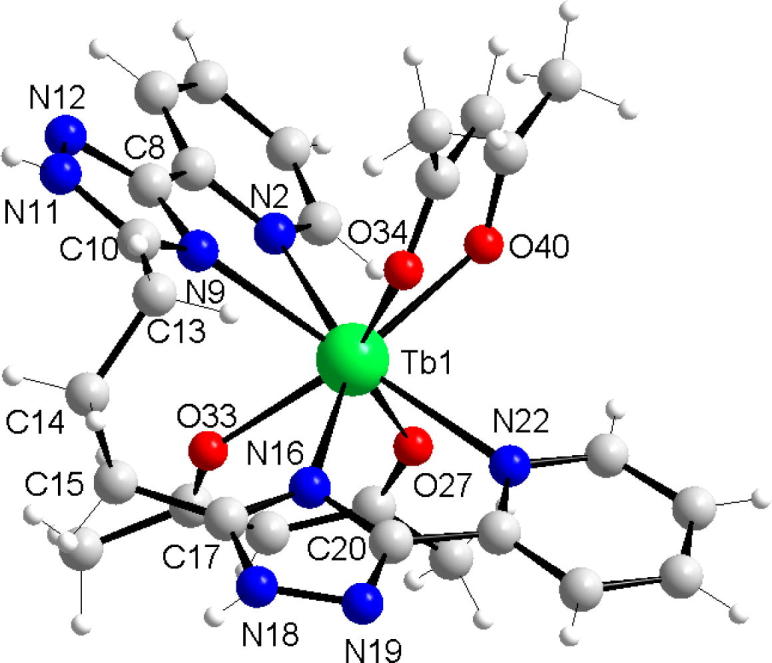
Molecular structure of the complex 1, counter chloride-anion is omitted for clarity.

Complex **1** crystallizes in monoclinic symmetry in the triclinic space group $P\overset{¯}{1}$. The obtained structure of cationic complex Tb(acac)_2_L^+^ is depicted in Fig. 1.

The central ion of Tb(III) is present in an eight-coordinate N_4_O_4_ environment, with the tetradentate bis(pyrindine-triazole) forming an equatorial ‘belt’ and the two bidentate acetylacetonate ligands occupying pseudo-axial positions. The coordination geometry of the metal center is best described as distorted square antiprism. The two “square” planes consist of atoms: O34/O40/N2/N9 (average deviation from the best-fit plane through them 0.025 Å) and O33/O27/N16/N22 (average deviation from the best-fit plane through them 0.055 Å) and form an angle 2.26°. The four N-donor atoms of L form an distorted square (average deviation from the best-fit plane through them, 0.396 Å), with Tb sitting out of this plane by 0.066 Å. Two coordinated pyridyl rings (containing N2 and N22 atoms) are inclined at an angle of 30.94° to each other. The Tb(III) is positioned out of the plane of the one acac^-^ ring, the angles between the planes defined by O–Tb–O and O–C–C–C–O atoms (where C and O atoms belong to a given 1,3-diketonato ring) is 25.83°, the remaining acetylacetonate chelating ring is almost planar and the angle mentioned above is 3.96°. The bond lengths between the terbium ion and the oxygen atoms of acac vary from from 2.277 to 2.352 Å. This is in agreement with expected Tb–O bond lengths in β-diketonates complexes. Slight asymmetry is observed for the bond lengths between Tb and nitrogen atoms of the triazole and the pyridine rings, respectively. The bonds lengths of the carbonyl groups are almost the same, indicating that a strong conjugation exists in the chelate rings.

In the unit cell are two complete and crystallographically independent cationic complexes; the lattice cavity is filled with two cyclohexane molecules, which are held by weak van der Waals forces. The crystal lattice is stabilized by hydrogen bonds between triazole rings and counter chloride-anion (N11–H⋯Cl50⋯N18–H) with complexes pair creation.

### 3.3 Photophysical properties

**Fig. 2:**
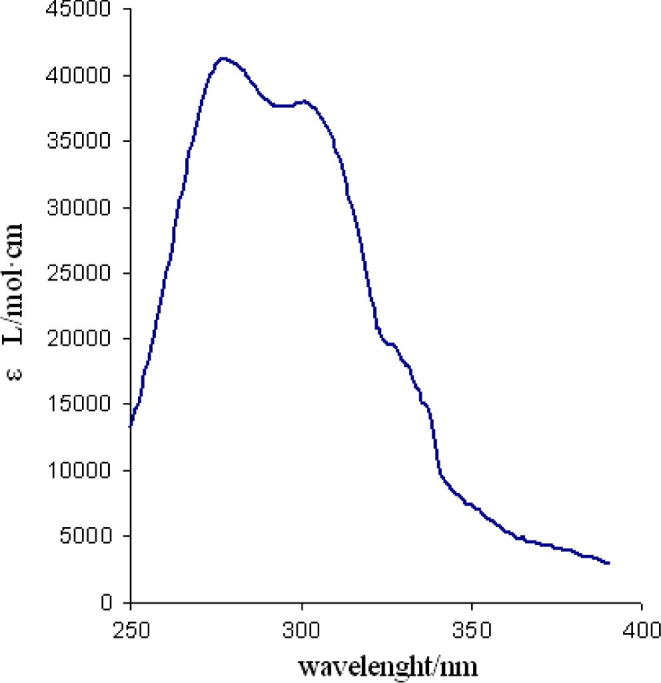
UV–Vis electronic absorption spectra of complex 1 recorded in MeOH solution.

The electronic absorption spectra for **1**–**4** were recorded in methanol solution at room temperature. Photophysical data of the investigated compounds are presented in the Table 2. UV–Vis spectra of the Ln(III) complexes within the spectral range of ligand-centered transitions were nearly identical, so, only the spectra of complex **1** are inserted in Fig. 2 as example.

**Table 2:** Photophysical properties of Ln complexes.

Compound	UV–Vis absorption *λ* (nm), *ε* (L/mol cm)	HST band (solid) *λ*_em_ (nm)	Quantum yield% solid/solution	Lifetime (solid) (μs)	Lifetime H_2_O (μs)	Lifetime D_2_O (μs)
**1**	279 (41200), 352 (37900)	612	38/7	1482	1034	1172
**2**	275 (41100), 352 (38100)	545	16/4	1092	608	878
**3**	277 (41200), 352 (37950)	645	4.5/–	51	–	–
**4**	275 (41200), 352 (38100)	575	0.7/–	17	–	–

All the Ln(III) complexes show an intense high-energy absorption band at about 274–279 nm (*ε* ∼41 200 L/mol cm) and a strong low-energy absorption band at about 300–308 nm (ε∼38 000 L/mol cm). The high-energy absorption band is assigned to a ππ^∗^ transition of the triazole ancillary ligand and the low-energy absorption band is attributed to the singlet–singlet ππ^∗^ transition of the acetylacetone ligand.

The visible luminescence spectra of the **1**–**4** upon UV excitation the complexes were recorded both in the solid state at 298 K and in water and MeOH solution and exhibit typical narrow band features. The ions Eu(III), Tb(III), Sm(III) and Dy(III) form a group of lanthanide whose complexes exhibit ionic luminescence in the visible region [12], [13]. Upon excitation UV-light title complexes exhibit photoluminescence in typical region. The excitation spectra of the **1**–**4** complexes were obtained by monitoring the characteristic emission of the corresponding Ln(III) ion. In the excitation spectra a broad band ranging from 270 to 410 nm is observed. The maximum of excitation spectra show features similar to the absorption spectra of the related complexes and are red-shifted by about 25–35 nm, thus indicating the indirect excitation by energy transfer from the ligand to the Ln(III) ion.

**Fig. 3:**
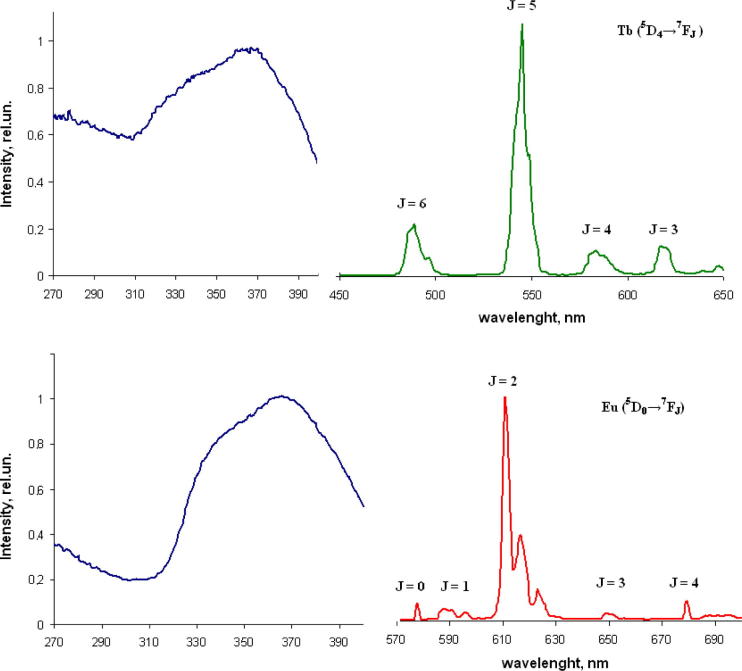
Normalized excitation and emission spectra of the Tb(III) and Eu(III) complexes recorded in solid state at 298 K excited at 365 nm. Excitation spectra are measured at the maximum of emission.

Luminescent and excitation spectra of Eu(III) and Tb(III) complexes in solid state are given in Fig. 3. The ligand-centered emission is not detected, suggesting an efficient ligand-to-metal energy transfer process. Upon excitation at 365 nm, the Eu complex exhibits the characteristic ^5^D_0_ → ^7^F*_J_* transition’s bands. The transition ^5^D_0_ → ^7^F_2_ (electric-dipole transition) at around 612 nm is the most prominent, rest ^7^F*_J_* (*J* = 0, 1, 3, 4) bands are not very intense. It should be noticed that hypersensitive transition ^5^D_0_ → ^7^F_2_ band presents three main components of fine structure, attributed to additional transitions as results Stark splitting. Same splitting we have found for relative complexes in our previous works [10]. The intensity ratio of the ^5^D_0_–^7^F_2_ transition and the ^5^D_0_–^7^F_1_ transition yields a value of 20.6. This data, as far as very low intensity of the ^7^F_0_ transition’s band are concerned, suggest that the Eu(III) ion exist in a low-symmetry point group.

The emission spectrum at 298 K in water and MeOH may also be interpreted as arising from metal ions and shows the same transitions and splitting (the spectrum becomes broader, as expected from a larger contribution from vibronic components due to flexibility of propylene-chain), pointing to a very similar structure in solution.

The [Tb(acac)_2_L]Cl complex, measured in the solid state at room temperature upon excitation at 365 nm, displays a typical emission spectrum with emission peaks centered at 489, 545, 584, 619 nm corresponding to the ^5^D_4_ → ^7^F*_J_* (*J* = 6, 5, 4, 3) transitions respectively. The ^5^D_4_ → ^7^F_6_ band is the most dominate one. No significant splitting of the transition bands were observed.

**Fig. 4:**
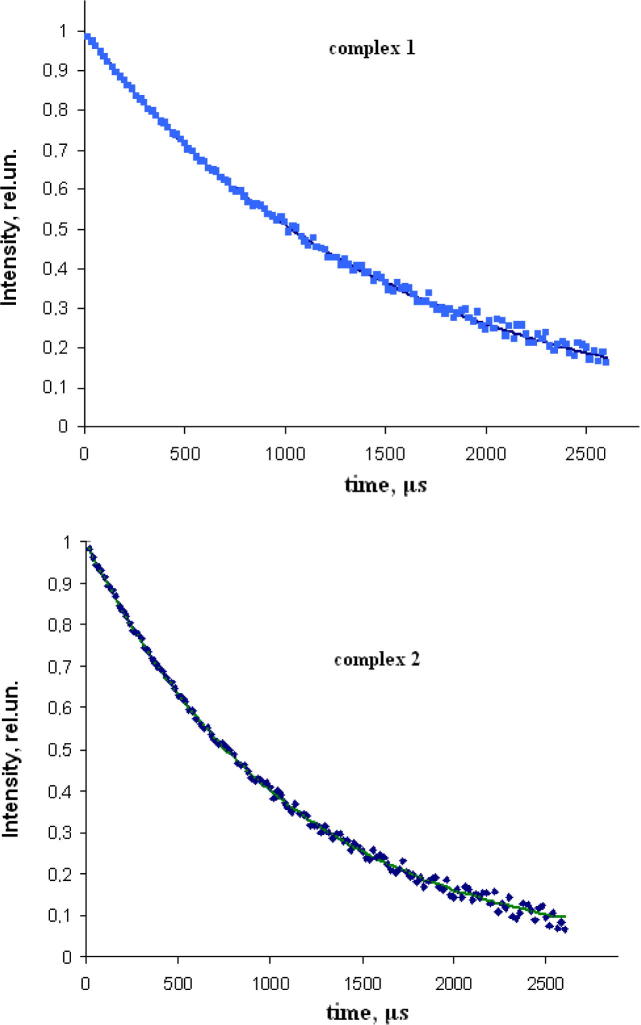
Luminescence decay profiles of Tb (monitored at 545 nm) and Eu complexes monitored at 612 nm measured in solid.

The decay curves of complexes **1** and **2** for ^5^D_4_ → ^7^F_5_ and ^5^D_0_ → ^7^F_2_ transitions respectively both in solid state and solutions were analyzed. The decay curves measured for solid samples are shown in Fig. 4. Both of the decay curves can be fitted by single exponential function. By fitting the luminescence lifetime of Tb and Eu are determined to be 1482 and 1092 μs, respectively. The luminescence lifetime measured in water solution of complexes **1** and **2**, are small and equals 1034 and 608 μs, respectively. Accordingly the decrease can be related to a rapid loss of excitation energy due to the conformational flexibility of propylene group or to the additional coordination of the water molecule and the loss of energy due to fluctuations of OH-oscillators. Although no solvent molecules were found in solid samples, coordination of the solvent molecules may well occur in solution. To determine this possibility the hydration numbers *q* were measured by comparison of the lifetimes in the water and D_2_O using the following equations [14], [15], [16]:For Tb $q=5(1/\tau_{{\text{H}}_{2}\text{O}}-1/\tau_{{\text{D}}_{2}\text{O}}-0.06)$, *q* = 0.21For Eu $q=1.2(1/\tau_{{\text{H}}_{2}\text{O}}-1/\tau_{{\text{D}}_{2}\text{O}}-0.25-0.075x)$, *q* = 0.26

The obtained results indicate the absence of solvent molecules in the first coordination sphere of the metal and are consistent with the presence of water molecules only in the secondary coordination sphere of the lanthanide complexes.

The luminescence efficiency of the Tb complex decreases from 38% in solid state to 7% in water. This is similar to the Eu complex **2** (which decreases from 16% in solid state to 4% in water). Good quantum yields in the solid state indicate efficient energy transfer from the ligand to the central atom. According to Latva’s empirical rule, optimal sensitizing for Tb(III) and Eu(III) luminescence requires an energy gap Δ*E*(*T*  − ^5^D_j_) in gate 2100–4500 cm^−1^ [17]. Since the energy of the triplet level of the acac-anion (25 310 cm^−1^) is located above the resonance level of the title lanthanide ions the complexes show emission. However, the Δ*E* values are 8051 and 4810 cm^−1^ for Eu(III) and Tb(III), respectively, and are significantly higher than the optimal value. The value of the triplet level of the ligand L was measured via the phosphorescence spectra of the gadolinium complex and is 22 989 cm^−1^. The coordination of L enhance the luminescence intensity of complexes **1** and **2** as a result of the cascade intramolecular energy transfer from the acetylacetonate-anion to L, then from L to Ln. The value of Δ*E* in this transition gets close to the optimal (5730 and 2489 cm^−1^ for Eu(III) and Tb(III), respectively). Therefore 1,3-bis(5-(pyridine-2-yl)-1,2,4-triazol-3-yl)propane acts as synergic agent, not only shielding against the influence of water molecules but strongly its promotes efficient energy transfer.

**Fig. 5:**
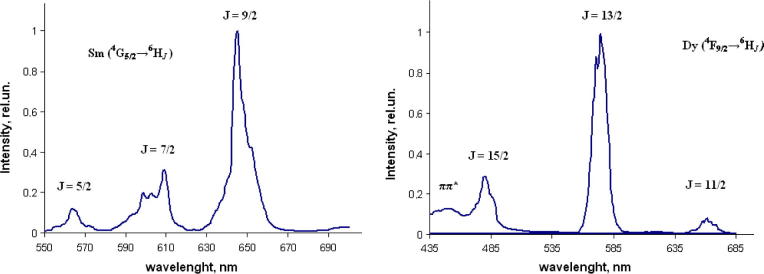
Normalized emission spectra of the Sm(III) and Dy(III) complexes recorded in solid state at 298 K excited at 365 nm.

Luminescence spectra of Sm(III) and Dy(III) complexes are shown in Fig. 5. Spectra have similar bands both in solid and in solution. Upon excitation at 365 nm the complex **3** shows three main peaks in luminescence spectrum observed at 565, around 605, and 645 nm, assigned to the ^4^G_5/2_ → ^6^H_J_ transitions, associated with *J* = 5/2, 7/2, 9/2 respectively. The transition ^4^G_5/2_ → ^6^H_9/2_ (electric-dipole transition) at around 645 nm is the most dominated. In the solid state spectrum the ^4^G_5/2_ → ^6^H_7/2_ band presents three main components of the fine structure, attributed to additional transitions resulting from Stark splitting of the term due to the low symmetry of the complex.

Solid samples of the Dy complex **4** exhibit the two characteristic emission lines of Dy(III) at 479 nm, 575 nm and weak emission band 658 nm, resulting from ^4^F_9/2_ → ^6^H*_J_* (*J* = 15/2, 13/2, and 11/2) transitions of the Dy(III) ion, respectively. There is a weak broad band centered at 450 nm registered as result of luminescence of the triazole ligand which proves ineffective energy transfer.

It is well known that the luminescence intensity of the pair Sm(III)/Dy(III) is considerably lower than that of the Eu(III)/Tb(III) ions since the probability of nonradiative deactivation of the excited states of these ions is considerably higher. The same trend is seen for the title complexes. The quantum yield measured for solid samples of **3** and **4** are 4.5% and 0.7% and are, as expected, much smaller than for **1** and **2**.

## 4 Conclusions

We have successfully designed new Tb(III), Eu(III), Sm(III) and Dy(III) complexes with bis(5-(pyridine-2-yl)-1,2,4-triazol-3-yl)propane and the acetylacetonate counter anion. Ion luminescence properties of the prepared complexes where studied both in solid and in solution. The bis(pyridine-triazole)-based ligand can efficiently promote the luminescence of Eu(III), Tb(III), Sm(III) and Dy(III) ions. This is associated with both, an optimal ratio of radiative levels of ligand and lanthanide ions and with successful shielding against the quenching influence of solvent molecules. Luminescent Ln(III) complexes appear to be a suitable candidates for enhancing color rendition of LED devices, and to be used as materials in telecommunications, bio-analyses, and medical bioprobes.
