# Supplementary material for: Synthesis, structure and luminescence studies of Eu(III), Tb(III), Sm(III), Dy(III) cationic complexes with acetylacetone and bis(5-(pyridine-2-yl)-1,2,4-triazol-3-yl)propane
Source: Inorganica Chim Acta. 2013 Sep 1;406:279–84. doi: 10.1016/j.ica.2013.04.006 (PMC3778742; doi:10.1016/j.ica.2013.04.006)
Supplement: Supplementary file 2 [file mmc2.zip › CCDC-Confirmation.pdf]

Alexey Gusev <galex0330@gmail.com> 18. Jänner 2013 22:40
An: Miki HASEGAWA <hasemiki@chem.aoyama.ac.jp>
Kopie: Wolfgang Linert <wlinert@mail.zserv.tuwien.ac.at>
Re: Inorg chim Acta

Dear Colleges!
I've deposited cif file before submission. Please see below confirmation data

Deposited Data - CCDC 917885
Dear Depositor

The following crystal structure has been deposited at the Cambridge
Crystallographic Data Centre and allocated the deposition number

CCDC 917885

If we have any queries relating to these data then we will contact you later.

Summary of Data CCDC 917885

Authors: Alexey Gusev

Journal:

Formula: C39 H44 Tb1 O4 N8 Cl1

Unit cell parameters: a 9.229(5) b 13.790(11) c 14.984(9)
alpha 86.64(5) beta 72.50(4) gamma 88.62(5)
space group P-1

Any publication carrying this CCDC number will refer to the
most recent set of deposited data; previous depositions carrying
this number will not be retained.

Please note, if data have not appeared in a journal publication
3 years after the date of deposition, and the CCDC cannot contact
you to discuss the matter, then the CCDC will automatically include
the data in the CSD as a Private Communication.

Kamila Nowakowska
Deposition Coordinator
CCDC
12 Union Road kamila@ccdc.cam.ac.uk
Cambridge Tel: +44 (0)1223 762911
CB2 1EZ
UK
www.ccdc.cam.ac.uk

2013/1/18 Miki HASEGAWA <hasemiki@chem.aoyama.ac.jp>:

> Dear Prof. Linert,
>
> I am asking my friend about the alert of CIF file.
> He was my assistant and moved to Toyama University now.
> Please wait a moment.
>
> Sincerely yours,
> Miki
>
> **
> 長谷川美貴
> 青山学院大学理工学部化学・生命科学科　教授
> 252-5258神奈川県相模原市中央区淵野辺5-10-1
> TEL +81-(0)42-759-6221, FAX +81-(0)42-759-6221,
> http://www.chem.aoyama.ac.jp/Chem/ChemHP/inorg2/
> --------------
> Dr. Miki HASEGAWA,
> Professor of Aoyama Gakuin University, Office:
> Department of Chemistry and Biological Science,
> College of Science and Engineering,
> Aoyama-Gakuin University,
> 5-10-1 Fuchinobe, Chuo-ku, Sagamihara,
> Kanagawa 252-5258, Japan
> **
>
> ---- Original message ----
>
>> Date: Fri, 18 Jan 2013 12:07:16 +0100
>> From: Wolfgang Linert <wlinert@mail.zserv.tuwien.ac.at>
>> Subject: Inorg chim Acta
>> To: Alexey Gusev <galex0330@gmail.com>, Miki Hasegawa <hasemiki@chem.aoyama.ac.jp>
>>
>> Dear Alexey,
>> dear Miki,
>>
>> I have uploaded most of the new Inrog chim paper. CCDC-confrimation is missing.
>>
>> Please have a look to the enclosed file for approval.
>>
>> all the best,
>>
>> Wolfgang

____
ICA-S-13-00055.pdf 905k バイト

--
Alexey Gusev
Assistant Professor
General Chemistry Department
V.I. Vernadsky Taurida national university
Simferopol, Crimea, Ukraine
+380504960295
e-mail galex0330@rambler.ru
